# Neurodevelopmental Effects of General Anaesthesia on the Developing Brain: A Structured Review of the Current Literature

**DOI:** 10.7759/cureus.99129

**Published:** 2025-12-13

**Authors:** Adnan Higgi, Carys Melvin

**Affiliations:** 1 Urology, Cardiff University, Cardiff, GBR; 2 Urology, Royal Glamorgan Hospital, Cardiff, GBR

**Keywords:** anaesthetic, cns, conditions impact academic performance, neuro development, pediatric

## Abstract

Concerns have emerged regarding the potential neurodevelopmental effects of general anaesthesia (GA) administered during early childhood. Recent research has investigated the long-term cognitive complications that may arise in children following the administration of GA. Preclinical studies suggest that anaesthetic agents may induce neuroapoptosis and disrupt neurogenesis in developing brains. This review evaluated current literature to determine whether GA exposure in children is associated with long-term cognitive or behavioural impairments.

A structured literature review was conducted using the PubMed database. The inclusion criteria focused on original research assessing neurodevelopmental outcomes for children following GA exposure. Retrospective cohort studies and prospective trials were analysed, while meta-analyses and systematic reviews were excluded. The retrospective cohort studies suggest that multiple or prolonged exposure to GA in childhood may be associated with increased risk of learning difficulties, behavioural disorders, and reduced academic performance. By contrast, the prospective studies, such as the general anaesthesia or awake-regional anaesthesia in infancy (GAS) trial, Paediatric Anaesthesia and Neurodevelopment Assessment (PANDA), and Mayo Anaesthesia Safety in Kids (MASK) trials, found no significant neurodevelopmental deficits following a single short-duration exposure. The MASK study did, however, note that potential behavioural risks were associated with repeated exposure. Variability in the design of the studies, the outcome measures, and confounding factors limited direct comparison across the studies.

The current evidence does not support a definitive link between single GA exposure and long-term neurodevelopmental harm in children but does suggest that repeated or prolonged exposure may increase the risk of harm. Further high-quality research is required to clarify these associations.

## Introduction and background

A 2015 article in BMJ Open identified 10 priority areas regarding anaesthesia that required further research. One of these areas is the potential for long-term cognitive complications to arise in children following the administration of general anaesthesia (GA) [1]. Preclinical data suggest that some anaesthetic agents may damage neurons in the developing brain. Apoptotic neurodegeneration has been observed in immature rodents, and common volatiles such as sevoflurane have been shown to cause defects in neurogenesis in rodents and primates [2,3]. Accordingly, the administration of GA to children has become an important area of research.

This structured narrative review explored the current literature to assess the understanding of the long-term cognitive effects of GA administration on children. The aim was to critically evaluate the existing research on the neurodevelopmental impact of GA on children and determine whether the evidence supports a causal relationship with long-term cognitive or behavioral deficits.

This work has not previously been presented, either in abstract form or as a full paper, at any major conference.

## Review

Methodology

This review was conducted in accordance with the Preferred Reporting Items for Systematic Reviews and Meta-Analyses (PRISMA) 2020 guidelines. A structured search of the PubMed database was performed by two independent reviewers. The search included studies that evaluated the association between the administration of a GA in childhood and long-term neurodevelopmental outcomes.

The search strategy was developed using the PubMed advanced search builder. It incorporated three key thematic components combined with Boolean operators, which are outlined in Table 1.

**Table 1:** The search strategy for the study majr: major; tiab: title/abstract; CNS: central nervous system

Line	Search Terms
#1	General anaesthesia [majr] OR Anaesthetic agents [majr]
#2	Developing brain [All Fields] OR CNS [tiab] OR Learning disability [tiab] OR Academic performance [tiab]
#3	Children [tiab] OR Infants [tiab] OR Childhood [tiab]
#1 + #2 + #3	General anaesthesia [majr] OR Anaesthetic agents [majr] AND Developing brain [All Fields] OR CNS [tiab] OR Learning disability [tiab] OR Academic performance [tiab] AND Children [tiab] OR Infants [tiab] OR Childhood [tiab]

**Figure 1:**
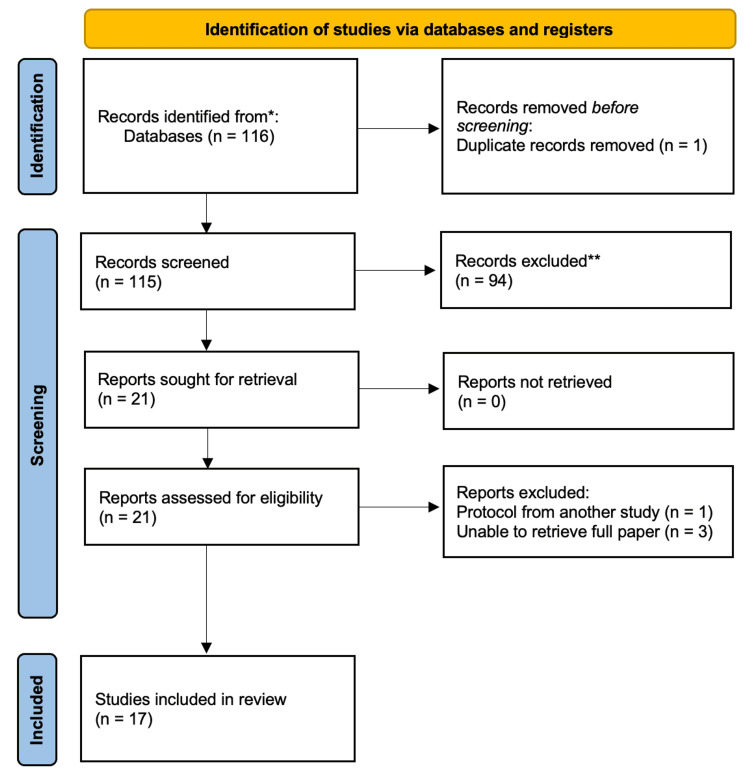
PRISMA chart PRISMA: Preferred Reporting Items for Systematic Reviews and Meta-Analyses

Consistent with the PRISMA 2020 standards, the eligibility criteria, data sources, and study selection process were clearly documented. The titles and abstracts were screened first, and then the full texts were reviewed to determine final inclusion. The study selection procedure is depicted in Figure 1, which presents a PRISMA 2020-compliant flow diagram. The extraction and analysis of the data were conducted in a manner consistent with the PRISMA recommendations, and the findings were organised systematically.

The aim of this study was to review primary research articles based on the inclusion and exclusion criteria described below. The initial search yielded a total of 116 articles for review, 1 of which was a duplicate.

Inclusion criteria

Studies that examined children aged 0-16 years who were exposed to GA and reported outcomes related to neurodevelopment were eligible for inclusion in the review. Only original research articles, including randomised controlled trials, cohort studies, and case-control studies, were included. Further, only articles that were published in English and available in full text were considered. Studies published after 2009 were prioritised to reflect modern anaesthetic practice.

Exclusion criteria

Studies that involved adult populations or animal-only models or that examined sedation or regional anaesthesia (RA) without a GA comparator were excluded from the review. Publications focusing solely on peri-operative physiological parameters without reporting long-term developmental outcomes were also excluded. Non-primary research articles such as reviews, meta-analyses, editorials, conference abstracts, and non-original research were automatically excluded. Studies without clearly defined neurodevelopmental outcomes were also excluded.

Data extraction

Filters were applied to include studies relating to the effects of the administration of GA on the developing brain. The titles and abstracts were screened for relevance, and then the full texts were assessed. The review followed the PRISMA 2020 guidelines and was conducted by two independent reviewers.

Of the 116 articles initially identified, 94 were excluded following the review of the titles and abstracts, and the one duplicate article generated by the search was removed. Another article was removed following full-text review because it was a protocol rather than an outcome study, and a further three articles were removed because it was not possible to retrieve the full report. The PRISMA chart presented in Figure 1 illustrates the search process.

Results

17 primary research articles were selected for final analysis and review. Table 2 presents the year, location, sample size, and design of each study.

**Table 2:** Summary of the articles reviewed for this study GA: general anaesthesia; RA: regional anaesthesia; GAS: general anaesthesia or awake-regional anaesthesia in infancy; PANDA: Pediatric Anaesthesia and Neurodevelopment Assessment; MASK: Mayo Anaesthesia Safety in Kids

Citation	Year	Location	Sample Size	Study Type/Analysis Method
Ing et al. [4]	2012	Australia	2,868	Retrospective cohort study
Ing et al. [5]	2017	Australia	1,622	Retrospective cohort study
Hu et al. [6]	2017	United States	1,136	Retrospective cohort study
Sprung et al. [7]	2012	United States	5,357	Retrospective cohort study
Wilder et al. [8]	2009	United States	5,357	Retrospective cohort study
Flick et al. [9]	2011	United States	4,684	Retrospective matched‐cohort study
DiMaggio et al. [10]	2011	United States	5,433	Sibling‐matched retrospective cohort study
Khochfe et al. [11]	2019	Lebanon	562	Comparative observational study (GA compared with RA)
Glatz et al. [12]	2017	Sweden	139,326	Population‐based cohort study
Block et al. [13]	2012	United States	577	Retrospective cohort study
Graham et al. [14]	2016	Canada	18,056	Retrospective cohort study
Davidson et al. [15] (GAS)	2016	Multinational	722	Randomized controlled equivalence trial
McCann et al. [16] (GAS)	2019	Multinational	722	Randomized controlled equivalence trial
Sun et al. [17] (PANDA)	2016	United States	105 sibling pairs	Sibling‐matched observational study
Warner et al. [18] (MASK)	2018	United States	997	Observational cohort study
Ko et al. [19].	2014	Taiwan	16,465	Retrospective matched cohort study
Hansen et al. [20].	2013	Denmark	17,264	Nationwide cohort study

Language and cognitive outcomes

Evidence from two observational cohort studies suggests that exposure to GA before age three may be associated with later deficits in language and abstract reasoning. Ing et al. reported that children exposed to GA before this age demonstrated lower receptive language scores (Clinical Evaluation of Language Fundamentals: Receptive (CELF-R)) (adjusted risk ratio, 1.87; 95% confidence interval (CI), 1.20-2.93) and cognition scores (adjusted risk ratio, 1.69; 95% CI, 1.13-2.53) compared with unexposed children even after a single GA exposure [4].

A follow-up investigation by Ing et al. found that exposure to GA for more than 35 minutes was associated with low total language scores [5]. The researchers used total language scores (Clinical Evaluation of Language Fundamentals: Total (CELF-T)) to assess the patients and divided the results into four quartiles. The cohort involved 148 individuals exposed to GA before age three and 96 with no documented exposure. The data showed that the CELF-T scores of the children in the first and second quartiles did not differ between those who were exposed to GA and those who were not. However, those in the third and fourth quartiles who were exposed to GA had lower scores (third quartile - unexposed: −5.3; 95% CI (−10.2 to −0.4); fourth quartile - unexposed: −6.2; 95% CI (−11.6 to −0.9)). These results indicated a potential relationship between language scores and the duration of anaesthesia, but an association with other medical comorbidities requiring prolonged anaesthetics and reduced language scores could not be excluded.

Academic performance: population-based evidence

Population-based evidence shows mixed outcomes in learning and academic achievement. Hu et al. performed a retrospective cohort analysis of 1,036 individuals (116 multiple exposures, 457 single exposures, and 463 unexposed) [6]. The results indicated that multiple anaesthetic exposures were associated with a higher risk of learning disability (hazard ratio (HR) for learning disabilities = 2.17; 95% CI, 1.32-3.59).

By contrast, Glatz et al. observed only a small reduction in academic performance associated with exposure to GA [12]. Their study involved 33,514 exposed children and 159,619 unexposed children. The data revealed a mean difference of 0.41% lower school grades (95% CI, 0.12-0.70%) among individuals who had received GA before age four. The findings included a small difference in academic performance, 0.79% (95% CI, 0.25-1.33%), in children who had been exposed to GA between the ages of 37 and 48 months. Similarly, Graham et al. reported no significant difference in kindergarten readiness scores following GA exposure (adjusted mean difference −0.02; 95% CI, −0.08 to 0.04; p = 0.52) [14].

Behavioral and developmental outcomes

Studies investigating behavioural outcomes suggest that multiple anaesthetic exposures may increase the likelihood of behavioral difficulties. Thus, DiMaggio et al. found an increased risk of developmental and behavioural disorders in a sibling-matched cohort of 10,450 children [10]. The incidence increased from 56.3 per 1,000 in the unexposed cohort to 128.2 per 1,000 in those exposed to a GA in early childhood (HR 1.6; 95% CI, 1.4-1.8). This risk also increased with multiple exposures.

Findings by Warner et al. from the Mayo Anaesthesia Safety in Kids (MASK) study involving 997 individuals demonstrated no significant difference in Intelligence Quotient (IQ) following a single exposure to GA [18]. The multiply and singly exposed children scored 1.3 points lower (95% CI, −3.8 to 1.2; p = 0.32) and 0.5 points lower (95% CI, −2.8 to 1.9; p = 0.70), respectively, than the unexposed children. However, the parents of children with multiple exposures reported higher rates of behavioural concerns than the parents of singly and unexposed children.

Khochfe et al. compared the outcomes of 226 children exposed to RA with those of 168 exposed to GA before the age of two [11]. Among these children, 12 in the RA cohort developed behavioural abnormalities compared with 44 in the GA cohort (p < 0.0001).

Evidence from prospective and randomised controlled studies

The prospective trial data indicate that a single brief exposure to GA has no clinically meaningful neurocognitive impact. Thus, in the general anaesthesia or awake-regional anaesthesia in infancy (GAS) trial, no significant difference in the cognitive composite scores between singly exposed and unexposed children was observed at age two [15]. The primary outcome of this trial was a mean difference of 0.2 points in the Bayley-III cognitive composite score between individuals exposed to RA and those exposed to GA (95% CI, −2.6 to 3.0).

Further, the evaluation by McCann et al. of neurodevelopmental outcomes for infants who underwent hernia repair with either brief GA (less than one hour of sevoflurane) or awake RA found no significant difference in neurodevelopmental outcomes between the groups at five years of age [16]. Specifically, the primary outcome of full-scale IQ measured using the third edition of the Wechsler Preschool and Primary Scale of Intelligence showed a mean difference of 0.23 IQ points between the GA and RA groups (95% CI, −2.59 to 3.06)

Similarly, the Paediatric Anaesthesia and Neurodevelopment Assessment (PANDA) sibling-matched study of 105 pairs found no statistically significant differences in the full-scale, performance, or verbal IQ scores after a single exposure to GA (full-scale IQ difference −0.2; 95% CI, −2.6 to 2.9) [17]. The very small mean IQ differences between the members of the pairs indicated that a single anaesthesia exposure in early childhood was not associated with measurable neurodevelopmental impairment.

Attention-deficit/hyperactivity disorder

A nationwide matched-cohort analysis by Ko et al. involving 16,465 children found no significant association between GA exposure in early life and subsequent attention-deficit/hyperactivity disorder (ADHD) diagnosis (HR 1.06; 95% CI, 0.86-1.31) [19]. These researchers reported that the adjusted HRs for developing ADHD following a single GA exposure compared with multiple exposures were 1.11 (95% CI, 0.88-1.41) and 0.96 (95% CI, 0.71-1.31), respectively.

Cohort studies of academic performance

Block et al. evaluated the academic outcomes of children exposed to GA and surgery during infancy [13]. The mean academic scores of the main cohort (n = 287) were significantly lower in those exposed to GA than in the overall population of the study (43.0 ± 22.4 for those exposed to GA compared with 50 for the overall population; p < 0.0001). 35 of the individuals in the exposed cohort (12%) scored below the fifth percentile (p < 0.00001).

A sub-study by Block et al. of medical records (n = 133) showed similar findings (45.9 ± 22.9; p = 0.0411) [13]. Among low-risk children with no neurological or medical comorbidities (n = 58), eight individuals (14%) scored below the fifth percentile (p = 0.008), though the mean scores did not differ from the expected scores (47.6 ± 23.4; p = 0.441). In this subgroup, the duration of anaesthesia correlated with poorer performance (r = −0.34; p = 0.0101; 95% CI, −0.55 to −0.08).

Hansen et al. compared Danish adolescents who underwent inguinal hernia repair in infancy (n = 2,689) with population controls (n = 14,575). After adjusting for demographic and parental factors, these researchers found the mean difference in grade-nine test scores to be minimal (−0.04; 95% CI, −0.09 to 0.01) [20]. The odds of non-attainment increased slightly (odds ratio (OR) 1.18; 95% CI, 1.04-1.35), though this increase was less when children with congenital anomalies were excluded (OR 1.13; 95% CI, 0.98-1.31).

Discussion

There is conflicting evidence in the literature regarding neurodevelopment and exposure to anaesthesia at a young age. Preclinical data show that anaesthetic agents cause neurodegeneration in animal models [2,3]. However, these data cannot necessarily be extrapolated to humans. Consequently, researchers have explored the risk associated with the administration of GA for neurodevelopment in children.

Various outcomes were measured in the various studies, mainly academic performance, neurocognitive tests, or clinical diagnoses of behavioral or learning difficulties in later life. Therefore, it is difficult to compare the results of the studies directly, but they do provide insights into the effects of anaesthesia on several cognitive domains in children.

Much of this research consists of retrospective cohort studies. While such studies can be useful for highlighting associations, they do not involve randomisation and may contain bias. This limitation increases the likelihood that confounding factors such as socioeconomic class, gender, or comorbidities explain the differences observed. Many of these studies also had relatively small sample sizes of exposed children.

The largest sample size among the studies involved more than 190,000 children, approximately 30,000 of whom had received GA before the age of four [12]. The results showed a weak association between GA and long-term academic performance. This study also exemplifies the imbalance between the number of participants in the case and control groups, an issue repeatedly seen throughout many studies. For example, two of the studies demonstrated adverse neurodevelopmental effects using language assessment as a measurable outcome, both of which were conducted on the same birth cohorts and used relatively small sample sizes, n = 321 and n = 148 [4,5]. In isolation, the usefulness of these studies for assessing the long-term effects of anaesthesia on children may be limited.

Three prospective studies, the GAS, PANDA, and MASK studies, have recently been concluded. The outcomes measured and the inclusion criteria varied slightly. None of the three demonstrated an increased risk of neurocognitive deficits following a single exposure to GA [15-18]. However, the MASK study did find that repeated exposure to GA may be associated with an increased risk of developing behavioural and learning difficulties [18].

The PANDA study analysed a sibling-matched cohort group, with one sibling having received a single GA prior to 36 months of age. This selection process minimised confounding factors such as genetic, educational, and social differences, thereby increasing the likelihood that exposure to GA could explain any differences [17]. However, the lack of blinding may have introduced bias, and the cohort mostly consisted of male siblings.

The MASK study used IQ testing as the primary outcome measure [18]. Because this was an observational study, it did not account for confounding factors. Also, selection bias may have been involved since not all of those invited to take part did so.

The GAS study was equivalent to a randomised controlled trial, for it measured the outcome (GA administration) against a control (spinal anaesthesia) [15,16]. This study may be more informative than the retrospective cohort studies, for it eliminated some of the selection bias through randomisation and reduced the confounding factors. Notably, no significant increase in risk was found between GA and RA, either during the initial results from the children at age two or when they were re-assessed at age five. There was the potential for bias involving the blinding of some parents regarding the intervention since parental reporting played a role in the outcomes.

Several studies also identified the duration of anaesthesia as a key prognostic indicator, in some cases suggesting that shorter exposure times were associated with fewer adverse effects [5,11,15,16]. A notable limitation of many of these studies is the failure to specify the doses or the anaesthetic agents administered. Therefore, it is difficult to determine whether any of the neurodegenerative effects assessed can be attributed to the dosage or the drug administered. The most recent of these studies included in the present review found that a single exposure was unlikely to affect a child’s neurodevelopment but suggested that cumulative exposure may do so [13].

A nationwide Danish study by Hansen et al. comparing children who had undergone inguinal hernia repair with a control group provided the reassuring result that there was no clear link between a single GA exposure and reduced academic performance in later life [20]. However, Block et al. concluded from their retrospective cohort study that a possible association may exist between early anaesthesia exposure and later academic performance, though confounding factors cannot be excluded [13].

Limitations of the review

Despite its comprehensive scope, this review is subject to several limitations. To begin with, significant heterogeneity among the included studies limited the ability to make direct comparisons between results. Further, many of the retrospective studies did not control for confounding variables such as socioeconomic status, parental education, and comorbidities that may have influenced neurodevelopmental outcomes independently. The wide variation in the sample sizes, with some studies relying on small cohorts of exposed children, resulted in reduced statistical power. Additionally, most of the studies did not specify the type, dose, or duration of the anaesthetic agents administered, so it is difficult to attribute the observed effects to specific pharmacological mechanisms.

Another limitation of this review is that the outcome measures were reported inconsistently across the studies. Thus, the reported outcomes included academic performance, behavioural diagnoses, and cognitive testing. This variation in the reported outcomes complicated the analysis. Several of the studies relied on parent-reported outcomes, and this approach introduces potential bias. Moreover, only a few of the studies involved extended follow-up into adolescence or adulthood, while the long-term effects were underreported in many of them. Lastly, the present review did not include a meta-analysis because of the heterogeneity of the studies included. Had it been feasible, such an analysis could have strengthened the quantitative interpretation of risk.

## Conclusions

This review highlights the complexity involved in assessing children’s neurodevelopmental outcomes following exposure to GA. The preclinical data from animal studies suggest that GA may be neurotoxic. However, human studies, particularly prospective trials, do not consistently support a causal link between a single GA exposure and long-term neurodevelopmental delay. Thus, the GAS, PANDA, and MASK studies provided reassuring evidence that a single exposure to a GA in early childhood is unlikely to be harmful, but retrospective studies continue to raise concerns about repeated or prolonged exposure. The main measurable outcomes affected related to academic performance and behavioral disorders.

To reach a definitive conclusion regarding the impact of GA exposure on children, future research must address the limitations in the current literature by conducting long-term follow-up extending into adolescence and adulthood. The reporting of detailed anaesthetic protocols, including the anaesthetic agent administered and the dosage, is necessary to provide more specific data. Additionally, there is a need to explore the genetic and environmental modifiers of risk, the dose-response relationships, and the cumulative exposure effects.

## References

[1] Boney O, Bell M, Bell N (2015). Identifying research priorities in anaesthesia and perioperative care: final report of the joint National Institute of Academic Anaesthesia/James Lind Alliance Research Priority Setting Partnership. BMJ Open.

[2] Yon JH, Daniel-Johnson J, Carter LB, Jevtovic-Todorovic V (2005). Anesthesia induces neuronal cell death in the developing rat brain via the intrinsic and extrinsic apoptotic pathways. Neuroscience.

[3] Lei S, Ko R, Sun LS (2018). Neurocognitive impact of anesthesia in children. Adv Anesth.

[4] Ing C, DiMaggio C, Whitehouse A (2012). Long-term differences in language and cognitive function after childhood exposure to anesthesia. Pediatrics.

[5] Ing C, Hegarty MK, Perkins JW (2017). Duration of general anaesthetic exposure in early childhood and long-term language and cognitive ability. Br J Anaesth.

[6] Hu D, Flick RP, Zaccariello MJ (2017). Association between exposure of young children to procedures requiring general anesthesia and learning and behavioral outcomes in a population-based birth cohort. Anesthesiology.

[7] Sprung J, Flick RP, Katusic SK (2012). Attention-deficit/hyperactivity disorder after early exposure to procedures requiring general anesthesia. Mayo Clin Proc.

[8] Wilder RT, Flick RP, Sprung J (2009). Early exposure to anesthesia and learning disabilities in a population-based birth cohort. Anesthesiology.

[9] Flick RP, Katusic SK, Colligan RC (2011). Cognitive and behavioral outcomes after early exposure to anesthesia and surgery. Pediatrics.

[10] DiMaggio C, Sun LS, Li G (2011). Early childhood exposure to anesthesia and risk of developmental and behavioral disorders in a sibling birth cohort. Anesth Analg.

[11] Khochfe AR, Rajab M, Ziade F, Naja ZZ, Naja AS, Naja ZM (2019). The effect of regional anaesthesia versus general anaesthesia on behavioural functions in children. Anaesth Crit Care Pain Med.

[12] Glatz P, Sandin RH, Pedersen NL, Bonamy AK, Eriksson LI, Granath F (2017). Association of anesthesia and surgery during childhood with long-term academic performance. JAMA Pediatr.

[13] Block RI, Thomas JJ, Bayman EO, Choi JY, Kimble KK, Todd MM (2012). Are anesthesia and surgery during infancy associated with altered academic performance during childhood?. Anesthesiology.

[14] Graham MR, Brownell M, Chateau DG, Dragan RD, Burchill C, Fransoo RR (2016). Neurodevelopmental assessment in kindergarten in children exposed to general anesthesia before the age of 4 years: a retrospective matched cohort study. Anesthesiology.

[15] Davidson AJ, Disma N, de Graaff JC (2016). Neurodevelopmental outcome at 2 years of age after general anaesthesia and awake-regional anaesthesia in infancy (GAS): an international multicentre, randomised controlled trial. Lancet.

[16] McCann ME, de Graaff JC, Dorris L (2019). Neurodevelopmental outcome at 5 years of age after general anaesthesia or awake-regional anaesthesia in infancy (GAS): an international, multicentre, randomised, controlled equivalence trial. Lancet.

[17] Sun LS, Li G, Miller TL (2016). Association between a single general anesthesia exposure before age 36 months and neurocognitive outcomes in later childhood. JAMA.

[18] Warner DO, Zaccariello MJ, Katusic SK (2018). Neuropsychological and behavioral outcomes after exposure of young children to procedures requiring general anesthesia: the Mayo Anesthesia Safety in Kids (MASK) study. Anesthesiology.

[19] Ko WR, Liaw YP, Huang JY (2014). Exposure to general anesthesia in early life and the risk of attention deficit/hyperactivity disorder development: a nationwide, retrospective matched-cohort study. Paediatr Anaesth.

[20] Hansen TG, Pedersen JK, Henneberg SW, Pedersen DA, Murray JC, Morton NS, Christensen K (2011). Academic performance in adolescence after inguinal hernia repair in infancy: a nationwide cohort study. Anesthesiology.

